# MINDMAP: establishing an integrated database infrastructure for research in ageing, mental well-being, and the urban environment

**DOI:** 10.1186/s12889-018-5031-7

**Published:** 2018-01-19

**Authors:** Mariëlle A. Beenackers, Dany Doiron, Isabel Fortier, J. Mark Noordzij, Erica Reinhard, Emilie Courtin, Martin Bobak, Basile Chaix, Giuseppe Costa, Ulrike Dapp, Ana V. Diez Roux, Martijn Huisman, Emily M. Grundy, Steinar Krokstad, Pekka Martikainen, Parminder Raina, Mauricio Avendano, Frank J. van Lenthe

**Affiliations:** 1 Department of Public Health, Erasmus MC, University Medical Center Rotterdam, P.O. Box 2040, 3000 CA Rotterdam, The Netherlands; 2 Research Institute of the McGill University Health Centre, Montreal, Canada; 3 Swiss Tropical and Public Health Institute, Basel, Switzerland; 4 University of Basel, Basel, Switzerland; 5 Department of Global Health and Social Medicine, King’s College London, London, UK; 6 Department of Epidemiology and Public Health, University College London, London, UK; 7 Inserm, UMR_S 1136, Pierre Louis Institute of Epidemiology and Public Health, Paris, France; 8 Sorbonne Universités, UPMC Univ Paris 06, UMR_S 1136, Pierre Louis Institute of Epidemiology and Public Health, Paris, France; 9 Epidemiology Unit, ASL TO3, Piedmont Region, Grugliasco, Turin, Italy; 10 Department of Clinical and Biological Science, University of Turin, Turin, Italy; 11 Geriatrics Centre, Scientific Department at the University of Hamburg, Hamburg, Germany; 12 Albertinen-Haus, Hamburg, Germany; 13 Drexel University Dornsife School of Public Health, Philadelphia, PA USA; 14 Department of Epidemiology and Biostatistics, EMGO+ Institute for Health and Care Research, VU University Medical Center, Amsterdam, The Netherlands; 15 Department of Social Policy, London School of Economics and Political Science, London, UK; 16 HUNT Research Centre, Department of Public Health and General Practice, Faculty of Medicine, Norwegian University of Science and Technology (NTNU), Levanger, Norway; 17 Levanger Hospital, Nord-Trøndelag Hospital Trust, Levanger, Norway; 18 Population Research Unit, Department of Social Research, University of Helsinki, Helsinki, Finland; 19 Canadian Longitudinal Study on Aging, Department of Health Research Methods, Evidence and Impact, McMaster University, Hamilton, Canada; 20 Department of Human Geography and Spatial Planning, Utrecht University, Utrecht, The Netherlands

**Keywords:** Ageing, Mental well-being, Urban health, Database, Data integration, Cohort studies

## Abstract

**Background:**

Urbanization and ageing have important implications for public mental health and well-being. Cities pose major challenges for older citizens, but also offer opportunities to develop, test, and implement policies, services, infrastructure, and interventions that promote mental well-being. The MINDMAP project aims to identify the opportunities and challenges posed by urban environmental characteristics for the promotion and management of mental well-being and cognitive function of older individuals.

**Methods:**

MINDMAP aims to achieve its research objectives by bringing together longitudinal studies from 11 countries covering over 35 cities linked to databases of area-level environmental exposures and social and urban policy indicators. The infrastructure supporting integration of this data will allow multiple MINDMAP investigators to safely and remotely co-analyse individual-level and area-level data.

Individual-level data is derived from baseline and follow-up measurements of ten participating cohort studies and provides information on mental well-being outcomes, sociodemographic variables, health behaviour characteristics, social factors, measures of frailty, physical function indicators, and chronic conditions, as well as blood derived clinical biochemistry-based biomarkers and genetic biomarkers. Area-level information on physical environment characteristics (e.g. green spaces, transportation), socioeconomic and sociodemographic characteristics (e.g. neighbourhood income, residential segregation, residential density), and social environment characteristics (e.g. social cohesion, criminality) and national and urban social policies is derived from publically available sources such as geoportals and administrative databases.

The linkage, harmonization, and analysis of data from different sources are being carried out using piloted tools to optimize the validity of the research results and transparency of the methodology.

**Discussion:**

MINDMAP is a novel research collaboration that is combining population-based cohort data with publicly available datasets not typically used for ageing and mental well-being research. Integration of various data sources and observational units into a single platform will help to explain the differences in ageing-related mental and cognitive disorders both within as well as between cities in Europe, the US, Canada, and Russia and to assess the causal pathways and interactions between the urban environment and the individual determinants of mental well-being and cognitive ageing in older adults.

**Electronic supplementary material:**

The online version of this article (10.1186/s12889-018-5031-7) contains supplementary material, which is available to authorized users.

## Background

From 1990 to 2010, the burden of mental ill-health increased by 38%, an increase mostly attributable to population ageing [1]. Mental disorders in old age lead to impairments in the ability to function socially, decreased quality of life, and increased risk of health problems and comorbidities. Poor mental well-being in later life carries significant social and economic impacts on families and societies, imposing a substantial burden on health and social care services [1]. Mental disorders associated with ageing, therefore, have become a key priority for public health policy and prevention.

Today, over 70% of Europeans and over 80% of North Americans reside in cities [2]. While urbanization is expected to increase in these regions over the coming decades, there is limited understanding of the critical contribution of the urban environment to mental well-being in ageing societies. Cities pose major challenges for older citizens, but also offer opportunities to develop, test, and implement policies, services, infrastructure, and interventions that promote mental well-being. The MINDMAP project, building on a novel database infrastructure, aims to identify the opportunities and challenges posed by urban environmental characteristics for the promotion and management of mental well-being and cognitive function of older individuals.

Funded from 2016 to 2020 by the Horizon2020 programme of the European Commission, MINDMAP aims to achieve its research objectives by bringing together ten longitudinal studies from eight European countries, the United States (US), Canada and Russia (in total over 35 cities of different sizes) linked to databases of area-level environmental exposures and social and urban policy indicators. Linking micro- (i.e. individual), meso- (i.e. neighbourhood), and macro- (i.e. city or national) level data enables MINDMAP to investigate the causal pathways and multi-level interactions between characteristics of the urban environment and the behavioural, social, and biological determinants of mental well-being and cognitive function in older adults.

Compared to studies based on a single country or city, integrating data from cohort studies in multiple cities offers many advantages for research exploring the impact of the urban environment on mental well-being. Harmonizing information across international cohort studies and combining them with data from different sources (physical, social and socioeconomic environmental characteristics, policy indicators) allows examining contextual determinants of variation in mental well-being across different populations and exploring the impact of neighbourhood, urban, and national policies for the prevention of mental disorders in older people. Furthermore, integrating data increases sample sizes and statistical power necessary to identify high-risk population subgroups, study relatively rare health conditions, unravel causal pathways and explore interactions between risk factors. Finally, and potentially most relevant for studies investigating environmental influences on health, integrating data from different geographical locations increases the variation in environmental characteristics and policies that influence mental well-being and cognitive function both within as well as between cities.

The MINDMAP database infrastructure will support these research objectives by integrating data from multiple sources and providing investigators with a platform to analyse it. The infrastructure will allow multiple MINDMAP investigators to safely and remotely co-analyse data from multiple sources and across different populations. Integration of different data sources will facilitate analyses exploring the importance of individual- and area-level determinants of mental well-being and cognitive function.

## Methods/design

### Participating institutions and cohort studies

Research centres and longitudinal cohort studies from across Europe and North America are involved in the MINDMAP consortium.

**Fig. 1:**
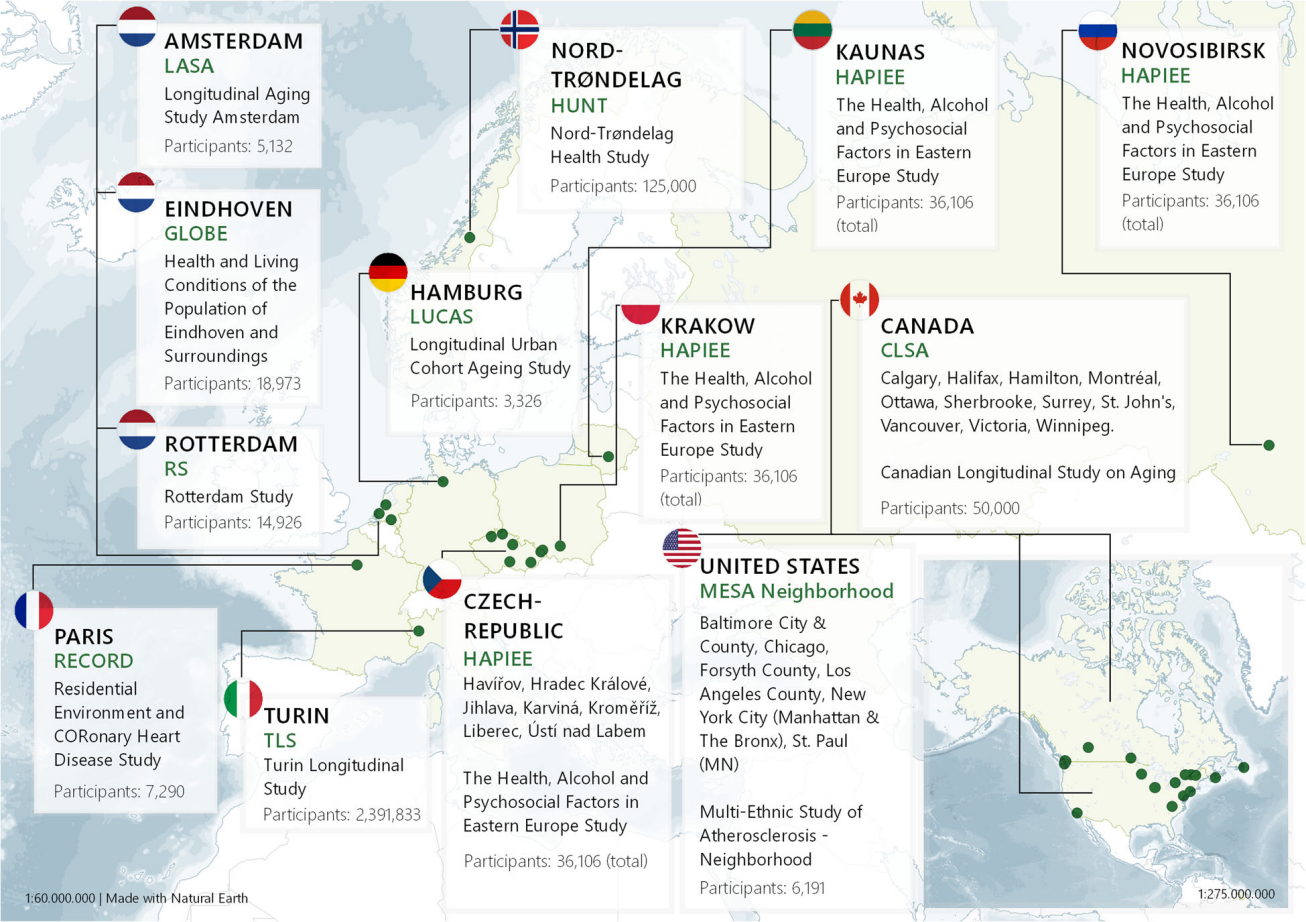
Overview of participating MINDMAP studies and their geographical locations

Thirteen research teams with a wide range of expertise are contributing to the MINDMAP project (see Additional file 1). MINDMAP also brings together ten ongoing longitudinal ageing cohort studies from eight European countries, the US, Canada, and Russia (Table 1). The European cohort studies appropriately cover urban areas in all regions including North, Central, Southern, and Eastern Europe (Fig. 1). Several cohort studies additionally include more rural areas, which will be useful for comparative purposes.

**Table 1:** Overview of MINDMAP participating cohort studies

Name	Type of study	Number of Participants	Country	Main study locations	Baseline	Last follow-up
Canadian Longitudinal Study on Aging (CLSA) [34]	Cohort	50,000	Canada	Victoria, Vancouver, Surrey, Calgary, Winnipeg, Hamilton, Ottawa, Montréal, Sherbrooke, Halifax, St. John’s	2008	Ongoing (until 2018)
Health and Living Conditions of the Population of Eindhoven and Surroundings (Gezondheid en Levens Omstandigheden Bevolking Eindhoven en omstreken; GLOBE) [35]	Cohort	18,973	Netherlands	Eindhoven and surroundings	1991	2016
The Health, Alcohol and Psychosocial Factors in Eastern Europe Study (HAPIEE) [36]	Cohort	36,106	Russia Poland Lithuania Czech Republic	Novosibirsk Krakow Kaunas Hradec Kralove, Jihlava, Karvina, Kromeriz, Liberec, Usti nad Labem	2002	2015
Nord-Trøndelag Health Study (HUNT) [37]	Cohort	125,000	Norway	Nord-Trøndelag county	1984	2008
Longitudinal Aging Study Amsterdam (LASA) [38]	Cohort	5132	Netherlands	West (including Amsterdam) East (including Zwolle) South (including Oss)	1992	2014
Longitudinal Urban Cohort Ageing Study (LUCAS) [39]	Cohort	3326	Germany	Hamburg	2000	2017
Multi-Ethnic Study of Atherosclerosis – Neighbourhood (MESA Neighbourhood) [7]	Cohort	6191	United States of America	Forsyth County (NC), Northern Manhattan & the Bronx (NY), Baltimore City & Baltimore County (MD), St. Paul (MN), Chicago (IL), Los Angeles County (CA)	2000	2012
Residential Environment and CORonary Heart Disease Study (RECORD) [40]	Cohort	7290	France	Paris	2007	2015
Rotterdam Study (RS) [41]	Cohort	14,926	Netherlands	Rotterdam (Ommoord)	1989	Ongoing (until 2020)
Turin Longitudinal Study (TLS) [42]	Registry based cohort	2,391,833	Italy	Turin	1971	2015

### Variables and data sources

MINDMAP is integrating data from numerous sources for different observational units. Individual-level data collected by longitudinal ageing studies will be combined with area-level urban characteristics and local and national policy indicators.

Additional file 2 provides a visual representation of the structure of the MINDMAP project, including all work-packages and their relation to the different data presented below. A detailed overview of data used in the MINDMAP project is provided in Additional file 3. The selection of variables was based on scientific literature and a draft conceptual model on the influence of environmental factors on mental well-being and cognitive function that is being developed by MINDMAP investigators.

#### Individual-level data

The MINDMAP consortium makes use of baseline and follow-up data collected by 10 participating studies.

##### Mental health, mental well-being and cognitive function

The main outcomes of interest within the MINDMAP project are indicators of mental health, mental well-being, and cognitive function. These indicators are measured in the cohort studies at multiple times through questionnaires, interviews, and cognitive tests and include variables covering life satisfaction, quality of life, depression and depressive symptoms, cognitive functioning, anxiety, and loneliness.

##### Individual-level determinants, mediators and confounders

MINDMAP-participating cohort studies have also collected detailed measures of sociodemographic variables, health behaviour characteristics, social factors, as well as measures of frailty and physical function indicators, and chronic conditions (multi-morbidities). An important feature of the MINDMAP studies is the collection of repeated measurement of determinants of mental well-being and cognitive function in cohort studies of urban residents. Several studies also have information available on blood derived clinical biochemistry-based biomarkers and genetic biomarkers.

#### Area-level data

Area-level information on physical environment characteristics (e.g. green spaces, transportation), socioeconomic and sociodemographic characteristics (e.g. neighbourhood income, residential segregation, residential density), and social environment characteristics (e.g. social cohesion, criminality) and national and urban social policies is derived from publicly available resources.

##### Physical environmental characteristics

Geospatial data is being collected from existing data portals, and city-specific contacts across the MINDMAP study sites. In the European Union, publicly available spatial information has drastically improved thanks to INSPIRE [3], a 2007 European Directive that establishes a data infrastructure for the collection and distribution of spatial information in the European Union. The European Data Portal [4] was systematically reviewed for all files containing items relevant to mental well-being or intermediary factors for all countries and cities of the participating European cohort studies. In addition, using the European Data Portal, relevant national, regional, and local data portals were identified and are systematically searched for relevant data that is not yet catalogued on the European Data Portal.

Harmonized high-resolution land use data, road infrastructure files, and residential address databases of the general population over the study territory were obtained for all European MINDMAP cities. For its land use data, MINDMAP extracted data from the European Urban Atlas [5]. This data is derived from satellite imagery and consists of 21 distinct categories, which capture a city’s land use (including public green areas). This data is being used to calculate individual ‘greenness’ exposure. In combination with the infrastructure information, measures such as nearest road network distance to urban green spaces are also being calculated. Point data of all residential addresses is used to determine population density. Information on facilities, transportation, and pollution have been obtained for a subset of cities from local and national data portals and are used to derive measures such as exposure to pollutants, access to public transport and availability of facilities.

The CLSA is part of the Canadian Urban Environmental Health Research Consortium (CANUE), a pan-Canadian initiative which is gathering and developing measures of environmental characteristics such as greenness, walkability, air pollution, and socioeconomic conditions for every neighbourhood across Canada [6]. As they become available, environmental characteristics developed within CANUE will be linked to CLSA cohort data. For our US cohort study, we will use the area-level geospatial data collected within the MESA neighbourhood study, which was specifically designed to study environmental influences on health [7].

##### Socioeconomic, sociodemographic and social environmental characteristics

Area level variables on neighbourhood socioeconomic measures (e.g. average income, proportion of rental housing), sociodemographic composition (e.g. proportion of older people, residential segregation), and social interaction indicators (e.g. proxies of social cohesion, criminality) are also being derived from publicly available sources such as the local and national statistics agencies and local governments.

##### National and local policies

Data on national and subnational policies that range from proximal to more distal influences on the mental well-being of older people in an urban environment has been collected within the MINDMAP project to evaluate the effects of public policies on mental well-being outcomes. Existing, cross-city and cross-national databases such as the Social Insurance Entitlements Dataset (SIED) [8], the Labour Market Reforms (LABREF) database [9], the Eurostat databases [10], and the OECD Long Term Care database [11] were the principal sources for social policies such as old age pensions and social care. Urban policy indicators, such as transportation affordability and accessibility indicators, were collected for each MINDMAP city from the Eurostat Urban Audit database [12] and the OECD Metropolitan Indicators database [13]. Mental health policy indicators, such as mental health system governance, resources and services were collected at the national level for European countries from the Eurostat Health Indicators database and the European Health for All database [14], and for all countries from the WHO Mental Health Atlas Country Profiles [15] and from two OECD data sources [16, 17]. MINDMAP aims to collate such policy data for the past 30 years, and earlier, when applicable. When longitudinal data was not available, we collected the latest available cross-sectional data. In addition, data has been collected on local mental health promotion and prevention policies through interviews with experts in MINDMAP cities [18].

### The MINDMAP process

**Fig. 2:**
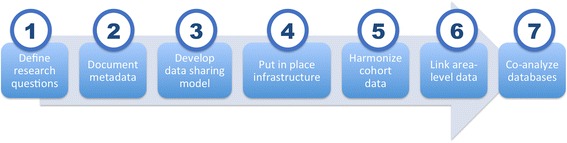
Step-by-step process to establish the MINDMAP integrated database infrastructure

To support cross-national research on ageing, mental well-being and the urban environment, the MINDMAP consortium adapted harmonization guidelines and software applications developed by Maelstrom Research [19, 20]. These tools have been employed under similar collaborative health research projects such as BioSHaRE [21], InterConnect [22], and the Canadian Partnership for Tomorrow Project [23]. Seven consecutives actions are being undertaken to establish an integrated database infrastructure allowing analyses of individual- and area-level data for research in ageing, mental well-being, and the urban environment (Fig. 2).

#### Define research questions

As a first step, MINDMAP consortium investigators identified a number of research questions addressing the variation in mental well-being and disorders in old age, both within cities as well as between cities and exploring how environments and policies at different levels might influence mental well-being in later life. Table 2 shows main research questions to be answered with the integrated database infrastructure. In addition, more detailed domain-specific research questions were defined, to be explored by each work package (Additional file 2).

**Table 2:** Main MINDMAP research questions to be answered with the integrated database infrastructure

1. How do variations across cities in mental well-being and cognitive outcomes in later life relate to urban environments, and how do they impact on co-morbidities?
2. How does the urban environment modify genetics and biomarkers as a potential mechanism through which features of the urban environment contribute to psychopathology in later life?
3. How do urban environmental characteristics influence mental well-being and cognitive outcomes in later life by shaping lifestyle behaviours?
4. How do psychosocial urban determinants influence mental well-being in later life?
5. How do ‘health-in-all’ and mental health prevention policies impact the mental well-being of older urban residents?

#### Document metadata

The design of participating studies and the data they collect were documented on a web-based platform [24]. This platform includes a search and query interface allowing MINDMAP investigators to quickly and easily identify studies collecting data items required to answer specific research questions. Questionnaires, standard operating procedures, and data dictionaries were also documented within the platform so that heterogeneity of data collection instruments could be properly assessed. Area-level urban characteristics as well as local and national policies of interest are also being documented.

#### Develop data sharing and publication guidelines

In order to establish basic governing principles for the consortium, MINDMAP principal investigators drafted guidelines covering access and usage of cohort study data and publication of results. First, each cohort study’s regular data access procedures will be respected, including the submission of access applications and obtainment of all required approvals from ethical review boards. Second, only data relevant to answer MINDMAP research questions is being requested. Third, after receiving all necessary approvals, these subsets of cohort study data will be hosted on firewall-protected servers. Participating studies were given the option of transferring a subset of their data to the coordinating centre’s (Erasmus MC) server or installing a local server at their home institution. Fourth, the MINDMAP coordinating team and cohort representatives will review each manuscript proposal. At this point, cohort representatives will need to confirm that they agree to the use of their data for a given manuscript, and will be able to opt-out if they wish. Lastly, a publication agreement was adopted to describe the authorship and acknowledgement guidelines relevant to work generated in connection with MINDMAP.

#### Put in place IT infrastructure

**Fig. 3:**
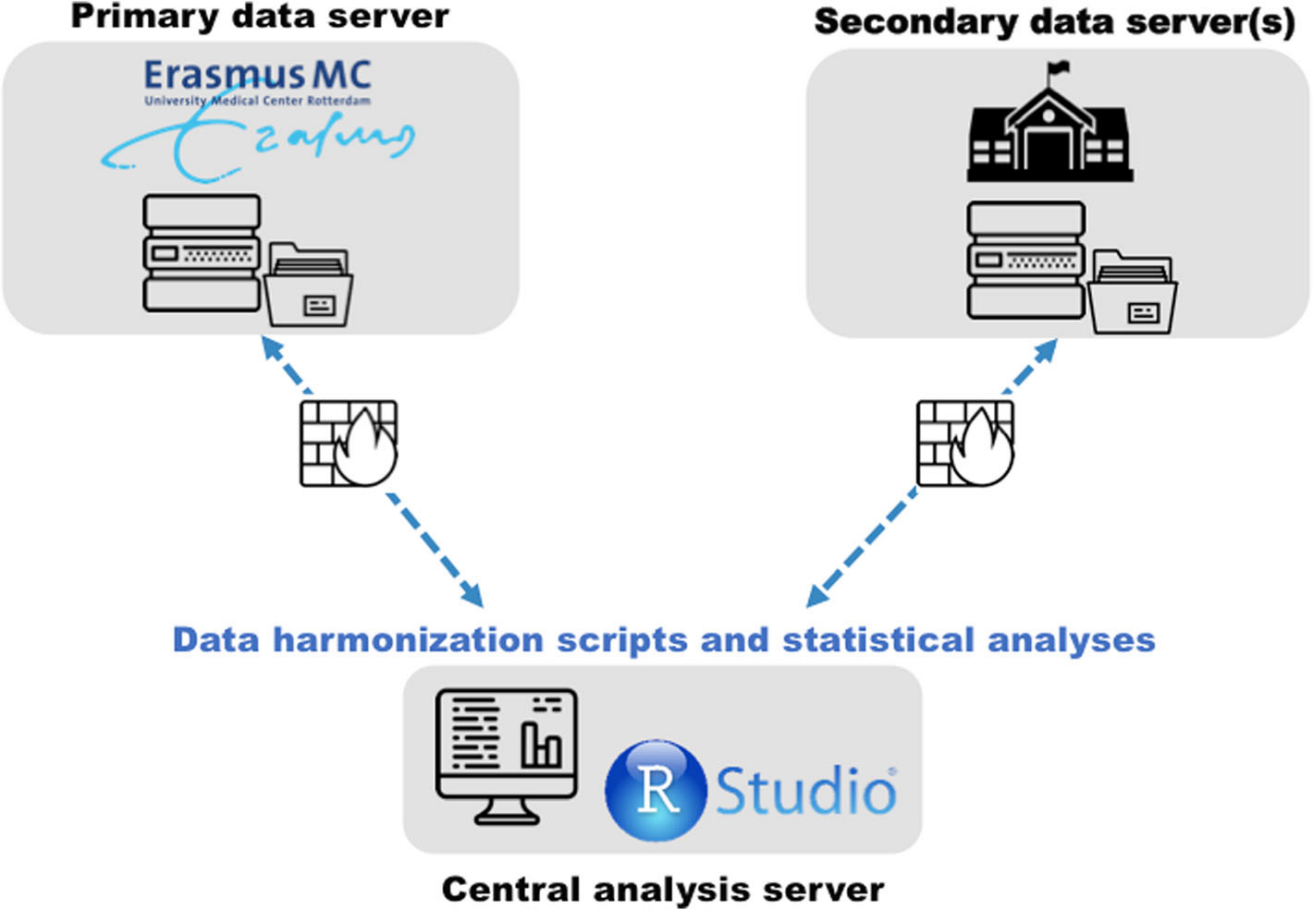
MINDMAP database infrastructure

Given potential restrictions related to sharing of individual-level data, a distributed database infrastructure was put in place to support data harmonization and cross-study analyses (Fig. 3). As such, a primary data server was installed at Erasmus Medical Centre in Rotterdam (the MINDMAP coordinating centre) to host datasets from studies whose policies allow the physical transfer of data to a third party. Cohort studies with more restrictive data sharing rules were given the option of installing secondary data servers in their own institution, which would be remotely accessible via encrypted connections (using HTTPS). Finally, a central analysis server running RStudio [25] was set up and allows authenticated MINDMAP staff and investigators to securely access firewall-protected data on the primary and secondary data servers (see step 7 below).

#### Harmonize cohort data

MINDMAP research teams were assigned specific domains of information to harmonize across all MINDMAP cohort studies. Assignment of data harmonization work was based on the expertise of the investigators at participating institutions. University College London is responsible for mental well-being and cognitive outcomes harmonization, Vrije Universiteit Amsterdam (VU) University Medical Centre was assigned social factors and perceived environment variables harmonization, Erasmus Medical Centre, in collaboration with McGill University Health Centre, is harmonizing socioeconomic variables, multi-morbidities and health behaviours variables. Finally, biomarker data is harmonized by McMaster University (for details on the domains of information, see Additional file 3).

Research teams began by reviewing the variables collected by each cohort study and related documentation (e.g. questionnaire(s), standard operating procedures, data dictionaries) for their assigned domain, and identifying missing information or highlighting unclear variable definitions, codes, or values. Targeted variables for harmonization are then defined (e.g. current cigarette consumption - categorical: yes (coded as 1) or no (coded as 0); pack-years of smoking - continuous variable) and documented in a central MINDMAP GitHub repository. The choice and specific definitions of targeted variables is determined by the research questions that they will help to address and the actual data collected by each cohort. Once defined, the potential for each cohort to generate target variables is assessed. Next, data harmonizers develop data transformation scripts to generate common-format variables in RStudio [25] on the password protected central analysis server. Decisions made and harmonization scripts applied for each study-specific dataset are documented using cohort-specific RMarkdown documents [26] in the publicly-accessible MINDMAP GitHub repository, thereby making data transformation decisions open and transparent. Lastly, quality control checks are conducted on harmonized variables by comparing the distribution and counts of harmonized datasets to the data originally collected by each study.

#### Link area-level data

**Fig. 4:**
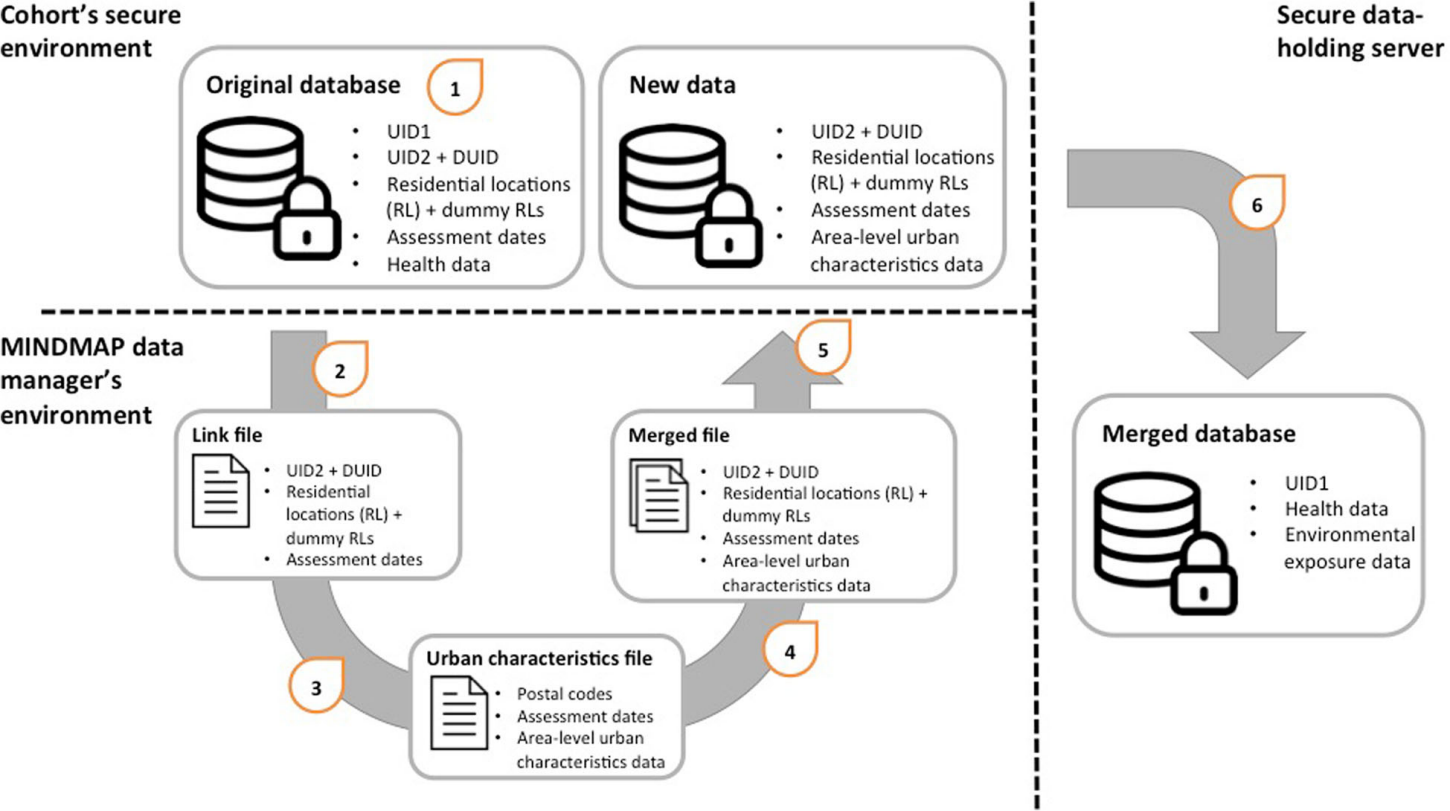
MINDMAP data linkage process

Addresses and postal codes of cohort participants will be used to link urban environmental characteristics and policy data (i.e. area-level data) to harmonized cohort data (Fig. 4). Given that the utilization of residential locations in research projects compromises study participants’ privacy, the georeferenced information will be blinded in a step-by-step process. Firstly, the cohort data manager will generate new unique identifiers (UID2) for all individuals in cohort studies along with dummy (i.e. random) identifiers (DUID) and residential locations (home address or postal code) for approximately 5% of the total cohort study’s sample (more if preferred). Second, a *Link file* containing UID2 and residential locations (RL) as well DUID and dummy RLs will be sent to the MINDMAP data manager. Third, MINDMAP will prepare a clearly documented *Urban characteristics file* to be merged with the *Link file*. Fourth, the *Link file* and *Environmental exposures* file will be merged into the *Merged file* using residential locations and dates of assessment. The resulting dataset is then sent back to the data manager of the cohort study who deletes all addresses. Lastly, the merged data is made available through the data infrastructure (either on the primary data server or a secondary data server).

#### Co-analyse integrated data

Using a web browser and secure internet connection, authenticated MINDMAP researchers can login to the central analysis server outlined in step four and conduct on-demand statistical analyses on geographically distributed firewall-protected databases using the RStudio web interface. While some studies have given permission for individual-level data to be analysed by MINDMAP investigators, others have restricted data access to aggregate-level information. For all analyses that include cohort studies prohibiting the use of individual-level data, the DataSHIELD approach is used [27, 28]. Under DataSHIELD, analysis requests are sent from the central analysis computer to the harmonized data held on the data servers. Computation is done simultaneously but in parallel on each data server linked by non-disclosive summary statistics. Individual-level cohort data thereby stay on their respective data server described in step four above.

Unlike experimental data, in our observational design, exposure to environmental and individual risk factors cannot be assumed to be randomly assigned [29, 30]. This is a challenge for research on the impact of the urban environment on health. To minimize risks of bias as much as possible with the available data, MINDMAP will capitalise on recent advances in causal inference and causal mediation methods, particularly derived from econometric and policy evaluation [29]. Because of the impossibility to randomize many of the key environmental determinants of mental well-being, quasi-experimental approaches applied to longitudinal data will provide the basis for the identification of causal effects. These techniques will include instrumental variables, regression discontinuity, and difference-in-differences approaches [31], which exploit naturally occurring changes in the environment, including policy reforms, to identify their causal effect on mental well-being. For example, the introduction of the free bus pass in England in 2006, a transportation policy, has been linked to increased physical activity and reduced obesity [32, 33]. Similar evaluations could be carried for the impact of policy reforms in the domains of housing, which affect the living arrangements of older people; pension policies, which influence the financial well-being of urban older dwellers; mental health promotion programmes that target the mental health of older people in cities; and environmental policies that affect access to outdoor and meeting spaces, lightening and walkability. MINDMAP will aim to implement policy evaluation studies to examine how some of these policies affecting older people living in MINDMAP cities may influence their mental health, with the aim of identifying transferrable lessons.

## Discussion

The MINDMAP project aims to identify the opportunities and challenges posed by the urban environment for the promotion of mental well-being and cognitive function in later life. MINDMAP aims to achieve its research objectives by bringing together longitudinal studies from 11 countries covering over 35 cities linked to databases of area-level environmental exposures and social and urban policy indicators. The infrastructure supporting integration of this data will allow multiple MINDMAP investigators to safely and remotely co-analyse individual-level and area-level data through a single platform.

The MINDMAP project has several important strengths. Integrating data from cohort studies in multiple cities and across various exposure or policy databases allows examining the role of contextual determinants on variations in mental well-being across different populations. It also increases variations across these contextual determinants and it raises sample sizes and statistical power and, because the data is pooled from different regions and jurisdictions, allows exploring the effect of policy on mental well-being. The harmonization approach and tools that are employed by the project have been methodically developed by Maelstrom Research [19, 20] and put to use in similar research collaborations [21–23]. These tools and approaches have been adapted to accommodate the specific needs of the MINDMAP project and ensure that all aspects of the harmonization project are carried out in a uniform, open, and methodical way to optimize the validity of the research results and transparency of the methodology. Moreover, the research teams contributing to the project bring a wide range of experiences and expertise that complement each other.

The integration of different data sources from different countries also present several challenges. Firstly, different questions and scales have been used within the participating cohort studies to measure similar underlying concepts. For some measures, harmonizing across the cohort studies is relatively straightforward (e.g. simple algorithmic transformations or calibrations). However, for measures such as mental well-being outcomes, this process is more complex, requiring the application of statistical modelling (e.g. standardization, latent variable or multiple imputation) [11]. Further, in many instances not all variables can be harmonized and constructed for all participating studies, because this might compromise the quality of the constructed variables. Secondly, all environmental data needs to be methodically checked for accuracy, completeness (e.g. missing roads), and geocoding or projection errors (e.g. a road is projected next to the real location of the road) to ensure the validity of the data. Furthermore, there is often a lack of historical data due to rapid changes in geographical information system (GIS) techniques and the tendency to only publish the most recent data by many of the sources publishing geospatial data. Extensive efforts are therefore needed to obtain high quality historical measures of environmental exposures. Thirdly, linking environmental data to cohort data can lead to privacy concerns when not dealt with properly. To prevent this, we developed a process to link the environmental data to cohort data that protects participant privacy by isolating residential addresses from privacy sensitive health data. Finally, integrating data from 10 longitudinal studies requires extensive coordination. Streamlining this process while respecting each study’s guidelines and regulations necessitates considerable time investments and meticulous planning.

MINDMAP is a novel research collaboration which is combining population-based cohort data with publicly available datasets not typically used for ageing and mental well-being research. Integration of various data sources and observational units into a single platform will facilitate multilevel analyses exploring the influence of individual- and area-level determinants of mental well-being. In the end, this infrastructure will help to explain the differences in ageing-related mental and cognitive disorders both within as well as between cities around the world and assess the causal pathways and interactions between the urban environment and the individual determinants of mental well-being and cognitive ageing in older adults.

## Additional files

Additional file 1: MINDMAP research teams. (DOCX 26 kb)

Additional file 2: Structure of the MINDMAP project. (DOCX 219 kb)

Additional file 3: Overview of data. (DOCX 31 kb)

## Abbreviations

CANUE: Canadian urban environmental health research consortium
CLSA: Canadian longitudinal study on ageing
DUID: Dummy unique identifier
GIS: Geographical information system
GLOB: Health and living conditions of the population of eindhoven and surroundings (Gezondheid en levens omstandigheden bevolking eindhoven en omstreken)
HAPIEE: The health, alcohol and psychosocial factors in eastern Europe study
HUNT: Nord-trøndelag health study (Helseundersøkelsen i Nord-Trøndelag)
LABREF: Labour market reforms
LASA: Longitudinal aging study Amsterdam
LUCAS: Longitudinal urban cohort ageing study
MESA: Multi-ethnic study of atherosclerosis
RECORD: Residential environment and CORonary heart disease study
RL: Residential locations
RS: Rotterdam study
SIED: Social insurance entitlements dataset
TLS: Turin longitudinal study
UID1: Unique identifier - original
UID2: Unique identifier - new
US: United States (of America)
VU: Vrije Universiteit Amsterdam
