# Supplementary material for: MINDMAP: establishing an integrated database infrastructure for research in ageing, mental well-being, and the urban environment
Source: BMC Public Health. 2018 Jan 19;18:158. doi: 10.1186/s12889-018-5031-7 (PMC5775623; doi:10.1186/s12889-018-5031-7)
Supplement: Supplementary file 1 — MINDMAP research teams. (DOCX 26 kb) [file 12889_2018_5031_MOESM1_ESM.docx]

***Annex 1: MINDMAP research teams***

The **Department of Public Health from the Erasmus MC in Rotterdam** is a leading centre of public health research both within the Netherlands, and internationally and it is the coordinating centre of the MINDMAP project. They have extensive experience with large European comparative studies and they are experts on environmental influences on health and health behaviour.

The **Department of Global Health and Social Medicine from King’s College London** in the United Kingdom, has world-leading expertise on global ageing, understanding the long-run impact of macro-economic shocks on the health of older people, and examining the health impact of social policies using longitudinal surveys and registry data.

**Maelstrom Research from Research Institute of the McGill University Health Center** in Montreal, Canada, is a leading expert on retrospective data harmonization and integration across studies. The methods developed by Maelstrom Research are applied and further improved within the MINDMAP project.

The **Department of Social Policy from the London School of Economics and Political Science**, in the United Kingdom, contributes unique knowledge on ageing research from a social perspective and can help to bridge the gap between research and policy.

The **Population Research Unit from the University of Helsinki** in Finland has wide-ranging experience with linking registry data, comparative studies, and areas effects on health.

The **Department of Epidemiology and Public Health from University College London** in the United Kingdom provides unique insight into understanding the determinants of health in Central and Eastern Europe and the former Soviet Union, in partnership with local investigators of the HAPIEE project in the Czech Republic, Russia, Poland and Lithuania.

The **Department of Public Health and Nursing from the Norwegian University of Science and Technology** offers vast expertise in large population studies and gene-environment interactions that benefit the MINDMAP project.

The **Department of Epidemiology and Biostatistics from the VU University Medical Center** is a leading centre of epidemiology of ageing and has a particular strong expertise on the social determinants of ageing.

**INSERM, the French National Institute of Health and Medical Research** in Paris is a work-leading institute on neighbourhood socioeconomic disparities in health and how multiple facets of the neighbourhood environment can influence health.

The **Geriatrics Research Department at the Albertinen-Haus and the University of Hamburg** are experts in geriatrics and functional ability within ageing population.

The **Department of Clinical Epidemiology and Biostatistics at McMaster University** in Hamilton, Canada has great knowledge on geroscience; the science to understand the processes of aging from cell to society.

The **Drexel University Dornsife School of Public Health** in in Philadelphia, Pennsylvania carries out world-leading research on social epidemiology by using novel methods in public health including agent based modelling.

The **Regional Epidemiology Unit ASL TO3 liaised with the University of Turin** in Italy has a wide expertise in the engagement of stakeholders at national and European level and in strategic communication with policy makers. They will help to valorise the results of the MINDMAP project.
