# Supplementary figures and images for: MINDMAP: establishing an integrated database infrastructure for research in ageing, mental well-being, and the urban environment
Source: BMC Public Health. 2018 Jan 19;18:158. doi: 10.1186/s12889-018-5031-7 (PMC5775623; doi:10.1186/s12889-018-5031-7)

***Annex 2: Structure of the MINDMAP project***


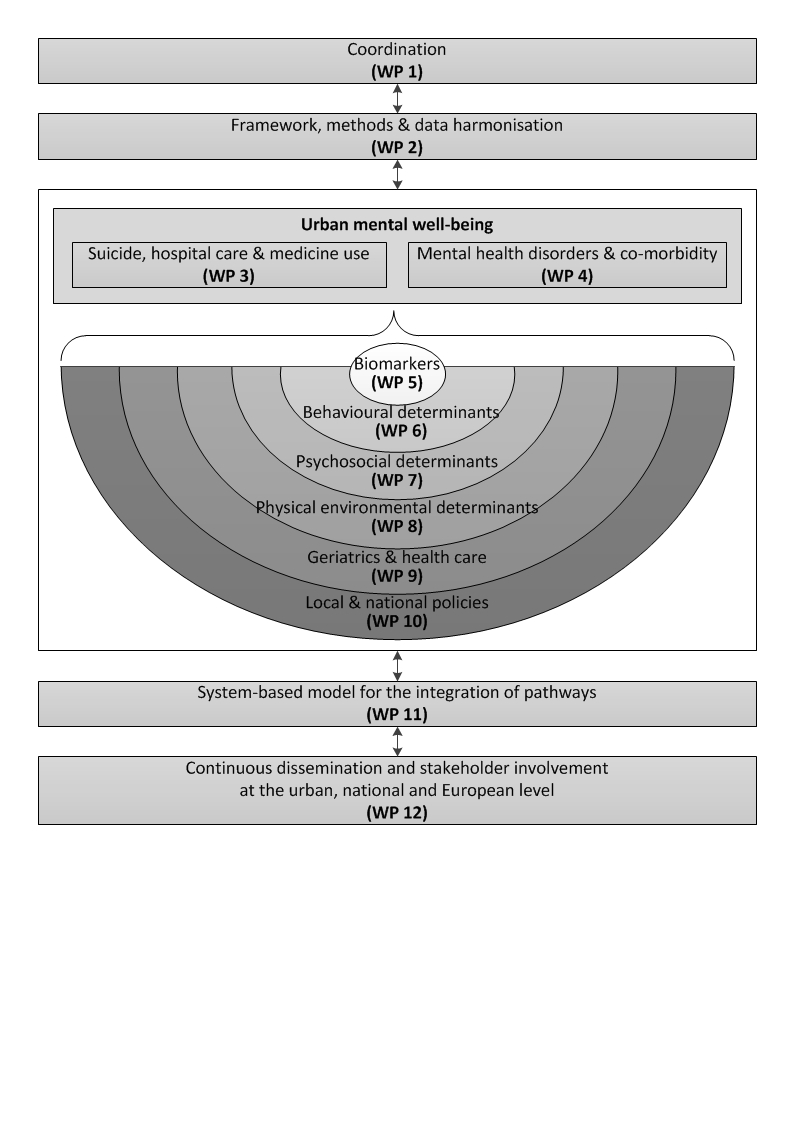


Note: WP = Work Package

Supplement: Supplementary file 2 — Structure of the MINDMAP project. (DOCX 219 kb) [file 12889_2018_5031_MOESM2_ESM.docx]
