# Supplementary material for: MINDMAP: establishing an integrated database infrastructure for research in ageing, mental well-being, and the urban environment
Source: BMC Public Health. 2018 Jan 19;18:158. doi: 10.1186/s12889-018-5031-7 (PMC5775623; doi:10.1186/s12889-018-5031-7)
Supplement: Supplementary file 3 — Overview of data. (DOCX 31 kb) [file 12889_2018_5031_MOESM3_ESM.docx]

***Annex 3: Overview of data***

| **Domain** | **Subdomains** | **Source** | **Level of measurement** |
| --- | --- | --- | --- |
| Mental health, well-being and cognitive outcomes | Life satisfaction, quality of life, depression and depressive symptoms, cognitive functioning, anxiety, loneliness | Cohort self-report and measurements | Individual |
| Sociodemographic variables | Age, sex, employment and retirement status, education, income, marital status, household structure | Cohort self-report and administrative data | Individual |
| Health behaviours | Alcohol consumption, tobacco use, diet and nutrition, physical activity, sleep quality | Cohort self-report | Individual |
| Social factors | Social and community support, social participation, social inclusion, major life event, home ownership | Cohort self-report and administrative data | Individual |
| Perception of urban environment | General neighbourhood safety, social trust, crime, social cohesion, deprivation | Cohort self-report | Individual |
| Other health outcomes (multi-morbidities) | Hypertension, Angina,  myocardial infarction, stroke,  BMI, perception of health,  disability, medication use | Cohort self-report and measurements | Individual |
| Biomarkers and genetics | Genetics, inflammatory markers, neuroendocrine markers, blood lipids, glucose, vitamins | Cohort biological samples | Individual |
| Built environment | Density, land use, infrastructure (roads, walking and cycling paths) | [European Environmental Agency](https://www.eea.europa.eu/data-and-maps/data/urban-atlas#tab-additional-information), national and subnational data portals | Small area |
| Local services | Public transportation proximity, (healthcare) facilities | National and subnational data portals | Small area |
| Pollution | Air pollution, noise pollution | National and subnational data portals | Small area |
| Neighbourhood socioeconomic position | Average neighbourhood income, proportion of rental houses, neighbourhood unemployment | National and subnational statistical agencies | Small area |
| Neighbourhood composition | Age composition, gender composition, residential segregation | National and subnational statistical agencies | Small area |
| Social-interaction indicators | Social cohesion, criminality | National and subnational statistical agencies & governments | Small area |
| Social policy indicators | Old age pensions, employment protections, housing & social care | Swedish Institute for Social Research [Social Insurance Entitlements Dataset](https://emea01.safelinks.protection.outlook.com/?url=http%3A%2F%2Fwww.edac.eu%2Fpolicies_desc.cfm%3Fv_id%3D202&data=01%7C01%7Cerica.reinhard%40kcl.ac.uk%7C3392095cea594333fbcf08d4ebacd3b7%7C8370cf1416f34c16b83c724071654356%7C0&sdata=cN5K%2FnP4Re1bJ13ftzYTjTmdhclFcLCtvmjwBTKZ19g%3D&reserved=0) (SIED), European Commission [Labour Market Reforms Database](https://webgate.ec.europa.eu/labref/public/) (LABREF), European Commission [Eurostat](http://ec.europa.eu/eurostat) Databases,  OECD [Long Term Care Database](http://www.oecd.org/els/health-systems/long-term-care.htm) | National |
| Urban policy indicators | Urban form, green spaces, transportation | OECD [Metropolitan Indicators Database](https://stats.oecd.org/Index.aspx?DataSetCode=CITIES), European Commission Eurostat [Urban Audit Database](http://ec.europa.eu/eurostat/web/cities/data/database) | City |
| Mental health policy indicators | Mental health system governance, resources & services, health insurances, promotion & prevention | World Health Organization [Mental Health Atlas Country Profiles](http://www.who.int/mental_health/evidence/atlas/profiles-2014/en/), World Health Organization European Office, European Health Information Gateway, [Health for All Database](https://gateway.euro.who.int/en/hfa-explorer/), European Commission [Eurostat Database, OECD](http://ec.europa.eu/eurostat/web/main/home) [Health Systems Characteristics Survey](http://qdd.oecd.org/subject.aspx?Subject=hsc)[, OECD](http://ec.europa.eu/eurostat/web/main/home) [Health Statistics](http://www.oecd.org/health/health-statistics.htm) | National |
